# Synergistic bactericidal effects of phage-enhanced antibiotic therapy against MRSA biofilms

**DOI:** 10.1128/spectrum.03212-23

**Published:** 2024-02-27

**Authors:** Ashlan J. Kunz Coyne, Kyle Stamper, Callan Bleick, Razieh Kebriaei, Susan M. Lehman, Michael J. Rybak

**Affiliations:** 1 Anti-Infective Research Laboratory, Department of Pharmacy Practice, Eugene Applebaum College of Pharmacy and Health Sciences, Wayne State University, Detroit, Michigan, USA; 2 Center for Biologics Evaluation and Research, US Food and Drug Administration, Silver Spring, Maryland, USA; 3 Department of Pharmacy Services, Detroit Receiving Hospital, Detroit Medical Center, Detroit, Michigan, USA; 4 Department of Medicine, Division of Infectious Diseases, Wayne State University, Detroit, Michigan, USA; Institut Pasteur, Paris, France

**Keywords:** MRSA, biofilm, bacteriophage, daptomycin, ceftaroline, bacterial synergy, bactericidal activity, medical device infections, phage-antibiotic combinations, *in vitro* models

## Abstract

Methicillin-resistant *Staphylococcus aureus* (MRSA) causes biofilm-related medical device infections. Phage-antibiotic combinations offer potential therapy due to proven *in vitro* antibiofilm efficacy. We evaluated phage-antibiotic synergy against biofilms using modified checkerboard and 24-h time-kill assays. Humanized-simulated daptomycin (DAP) (10, 8, and 6 mg/kg q24h) and ceftaroline (CPT) (600 mg q12h) were combined with Intesti13, Sb-1, and Romulus phages (tMOI 1, q12h). Assays were conducted in 168-h biofilm reactor models against DAP non-susceptible (DNS) vancomycin intermediate *S. aureus* (VISA) MRSA D712 and DAP-susceptible MRSA 8014. Synergistic activity and bactericidal activity were defined as ≥2log_10_ CFU/mL reduction from antibiotic-only regimens and ≥3log_10_ CFU/mL decrease from baseline at 24 h. Differences were analyzed by one-way analysis of variance with Tukey’s *post hoc* test (*P* ≤ 0.05 is considered significant). Surviving bacteria were examined for antibiotic minimum biofilm inhibitory concentration (MBIC) changes and phage susceptibility. In 168-h biofilm models, humanized DAP 10 mg/kg + CPT, combined with a 2-phage cocktail (Intesti13 + Sb-1) against D712, and a 3-phage cocktail (Intesti13 + Sb-1 + Romulus) against 8014, demonstrated synergistic bactericidal activity. At 168 h, bacteria were minimally detectable [2log_10_ CFU/cm^2^ (−Δ4.23 and −Δ4.42 log_10_ CFU/cm^2^; both *P* < 0.001)]. Antibiotic MBIC remained unchanged compared to baseline across various time points. None of the tested bacteria at 168 h exhibited complete phage resistance. This study reveals bactericidal efficacy of DAP + CPT with 2-phage and 3-phage cocktails against DNS VISA and MRSA isolates (D712 and 8014) in biofilm models, maintaining susceptibility. Further research is needed for diverse strains and durations, aligning with infection care.

**IMPORTANCE:**

The prevalence of biofilm-associated medical device infections caused by methicillin-resistant *Staphylococcus aureus* (MRSA) presents a pressing medical challenge. The latest research demonstrates the potential of phage-antibiotic combinations (PACs) as a promising solution, notably *in vitro* antibiofilm efficacy. By adopting modified checkerboard and 24-h time-kill assays, the study investigated the synergistic action of phages combined with humanized-simulated doses of daptomycin (DAP) and ceftaroline (CPT). The results were promising: a combination of DAP, CPT, and either a 2-phage or 3-phage cocktail effectively exhibited bactericidal activity against both DAP non-susceptible vancomycin intermediate *S. aureus* MRSA and DAP-susceptible MRSA strains within 168-h biofilm models. Moreover, post-treatment evaluations revealed no discernible rise in antibiotic resistance or complete phage resistance. This pioneering work suggests the potential of PACs in addressing MRSA biofilm infections, setting the stage for further expansive research tailored to diverse bacterial strains and treatment durations.

## INTRODUCTION

Biofilms have great clinical relevance given that biofilm formation is involved in an estimated 65%–80% of infections and often involve an indwelling medical device (1–3). Indwelling medical devices are particularly susceptible to bacterial biofilm formation due to their unique surface structures, making them excellent supports for bacterial adhesion (4). Biofilms are structured communities of bacteria embedded in an extracellular polymeric matrix that confers survival benefits to bacteria against host defenses and antimicrobial agents due, in part, to delayed antimicrobial penetration through the biofilm matrix and an altered organism growth rate (5, 6).

Methicillin-resistant *Staphylococcus aureus* (MRSA) is the leading cause of hospital-acquired infections and among the most common etiological agents of biofilm-mediated prosthetic medical device infections in the U.S. (7, 8). The rapid development of MRSA drug resistance and the formation of biofilms seriously challenge the clinical application of standard of care (SOC) antibiotics. Bacteriophages (phage) are obligate intracellular parasites of bacteria that undergo coevolution with their hosts in a dynamic milieu, wherein phages strive to outmaneuver bacterial defenses while bacteria employ sophisticated mechanisms to elude phage predation (9). This coevolutionary arms race makes phage an attractive option for treating clinically relevant bacteria, given their demonstrated *in vitro* activity against bacteria that have developed antimicrobial resistance due to acquisition or association with biofilms (10). Limited studies have reported the ability of phages to target biofilms, including those caused by multidrug-resistant *S. aureus* clinical isolates (11–13). In this regard, phage therapy may be useful in the setting of biofilm-associated infections, especially in patients unable to undergo source control due to detrimental consequences including loss of function, surgical risk, or a reduced quality of life (e.g., a left ventricular assist device or prosthetic joint that cannot be removed). The mechanism behind this antibiofilm activity is thought to be associated with enzymes such as depolymerases and lysins, which bind to polysaccharides and peptidoglycan, respectively (14). Other proposed mechanisms include endolysin-mediated downregulation of bacterial autolysin, inhibition of quorum sensing, and activation of stress responses (15).

The use of antibiotics in combination with phage is hypothesized to enhance the efficacy of phage-only treatment by targeting bacterial cells that may not be susceptible to phage infection alone (13, 16). Additionally, antibiotics can disrupt bacterial metabolism and weaken the biofilm structure, increasing the accessibility of phages to their targets (17). Furthermore, the use of antibiotics can potentially limit the emergence of phage-resistant mutants by exerting selective pressure on the bacterial population (13, 16, 18). Antibiotics combined with multiple phages in a phage cocktail, rather than a single phage, offer potential benefits in combating MRSA biofilms. First, MRSA strains can exhibit genetic diversity and develop resistance to specific phages over time (19). By employing multiple phages in a cocktail, the likelihood of encountering phage-resistant strains can be reduced, ensuring a broader spectrum of stable activity (13). The combination approach of antibiotics with phage cocktails also offers a synergistic effect, maximizing the chances of successful biofilm eradication and overcoming the challenges posed by MRSA biofilm infections (20).

Previously, we detailed phage selection studies conducted to assemble a phage cocktail composed of phages that demonstrate a broad host range against diverse *S. aureus* and were least prone to cross-resistance (13). The resultant phage cocktail containing phages Intesti13, Sb-1, and Romulus, when combined with standard of care antibiotics used to treat serious MRSA infections, demonstrated potent synergistic effects against MRSA biofilms that exhibited significant reductions in bacterial viability, effectively eradicating biofilm in static conditions (13). Furthermore, we identified that the presence of the 3-phage cocktail in phage-antibiotic combination (PAC) maintained antibiotic and phage susceptibility. Until clinical trials are generated to evaluate specific PAC efficacies, it is valuable to study such combinations in surrogate dynamic settings that recapitulate human-like pharmacokinetics/pharmacodynamics (PK/PD) antibiotic dosing.

Leveraging the results and methodology of the aforementioned study, the studies herein aimed to systematically investigate the PAC with standard of care antibiotics. The antibiotic dosing was designed to achieve antibiotic PK/PD exposures that are humanized over an extended period of time in 168-h PK/PD biofilm models against clinical MRSA strains D712 and 8014. This evaluation sought to assess the PACs with maximal bactericidal activity and its potential in preventing and/or reversing antibiotic and phage resistance.

## RESULTS

### Biofilm modified checkerboard assay results

**Fig 1:**
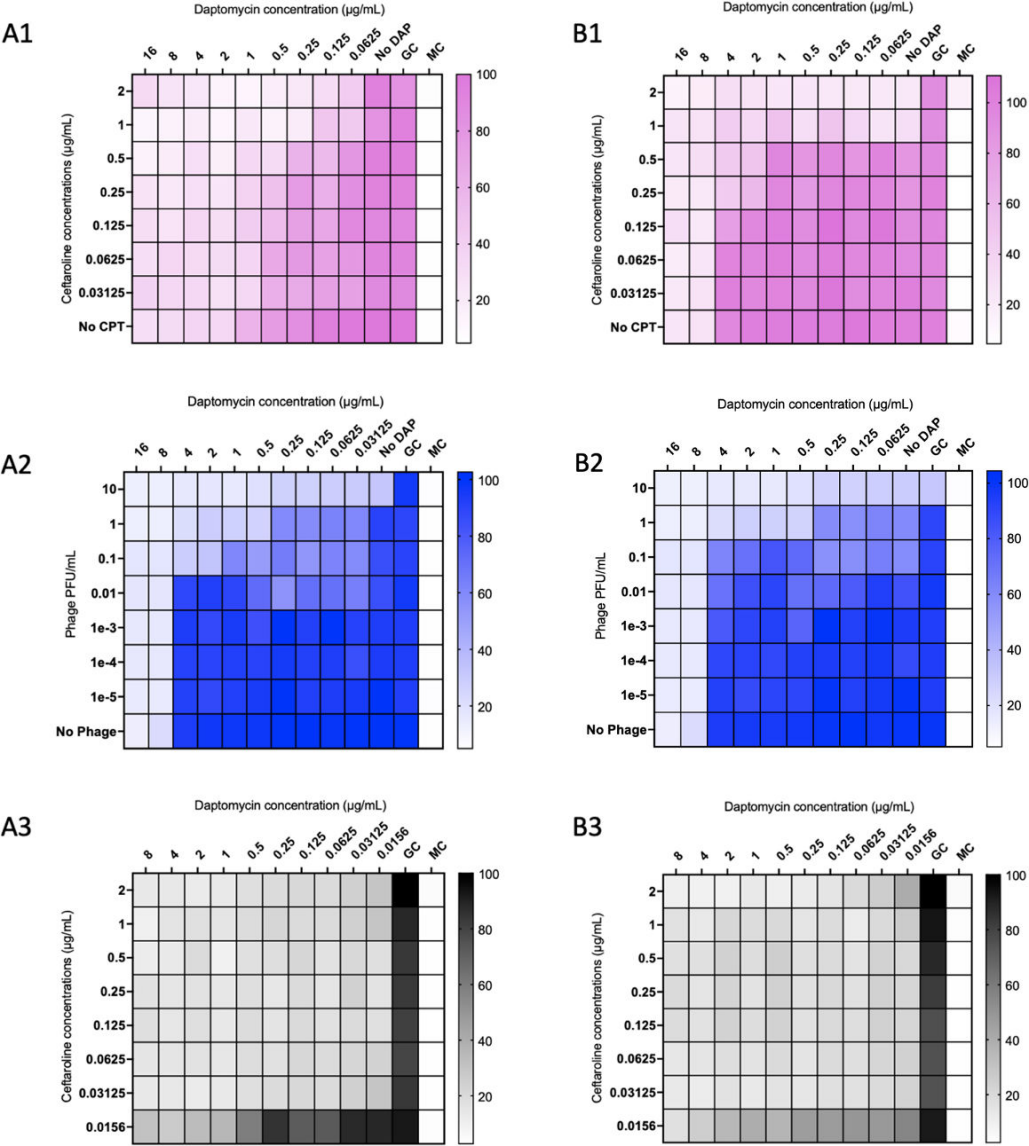
Modified checkerboard MBIC assay of MRSA isolates (**A**) D712 and (**B**) 8014 against (**A1, B1**) daptomycin plus ceftaroline, (**A2, B2**) daptomycin plus the 2-phage or 3-phage cocktail, and (**A3, B3**) daptomycin plus ceftaroline plus the 2-phage or 3-phage cocktail. Phage was added to each non-GC and non-MC well in (A3) and (B3) at a phage tMOI of 0.1. Values were normalized to the media control and converted to percent growth versus the untreated growth control. MOI, multiplicity of infection; DAP, daptomycin; CPT, ceftaroline; PFU, plaque-forming units; GC, growth control; and MC, media control.

To evaluate phage-antibiotic synergy (PAS) and identify the optimal phage theoretical multiplicity of infection (tMOI) in PAC prior to 24-h time-kill analyses (TKA) and 168-h *in vitro* biofilm models, bacterial growth was assessed against dilutions of daptomycin (DAP) and/or ceftaroline (CPT) and phage titer using modified checkerboard (CB) minimum biofilm inhibitory concentration (MBIC) microtiter plate assay in which the optical density of the culture in each well was measured following 24 h of incubation (Fig. 1). Spectrophotometric data were converted to a heat map of percent growth compared to growth control (21). Because this assay measures optical density of the media surrounding biofilms, it was not a direct measure of anti-biofilm activity. However, it was useful as a high-throughput screen to help select assay parameters for the subsequent TKA and PK/PD models, which do directly measure biofilm reduction.

We tested two *S*. *aureus* strains, D712 [DAP non-susceptible vancomycin intermediate *S. aureus* (DNS VISA)] and 8014 (MRSA), in modified CB MBIC (representative data shown). The 2-phage cocktail containing Intesti13 + Sb-1 was combined with DAP or DAP + CPT in selected D712 CB MBIC assays while the 3-phage cocktail containing Intesti13 + Sb-1 + Romulus was added to selected DAP and DAP + CPT CB MBIC for 8014. The selection of 2-phage versus 3-phage cocktail for use in CB MBIC against D712 and 8014, respectively, was based on previously reported data demonstrating retained phage susceptibility in TKA for 8014 with the 3-phage but not 2-phage cocktail (13). For D712, phage susceptibility was retained with the use of either 2-phage or 3-phage cocktail (13). In CB MBIC that contained DAP + CPT and a phage cocktail, a standard tMOI of 0.1 [2 × 10^8^ plaque-forming units (PFU)/mL] for each phage was added to appropriate wells. The combination of DAP + CPT without the addition of phage demonstrated additive activity with a fractional inhibitory concentration (FIC) of 1 (Fig. 1A1 and B1). DAP combined with the 2-phage and 3-phage cocktails demonstrated additive activity with an FIC of 1 (Fig. 1A2 and B2). The combination of DAP + CPT with the 2-phage and 3-phage cocktails demonstrated synergistic activity with an FIC of 0.5 (Fig. 1A3 and B3).

### Time-kill analysis results

**Fig 2:**
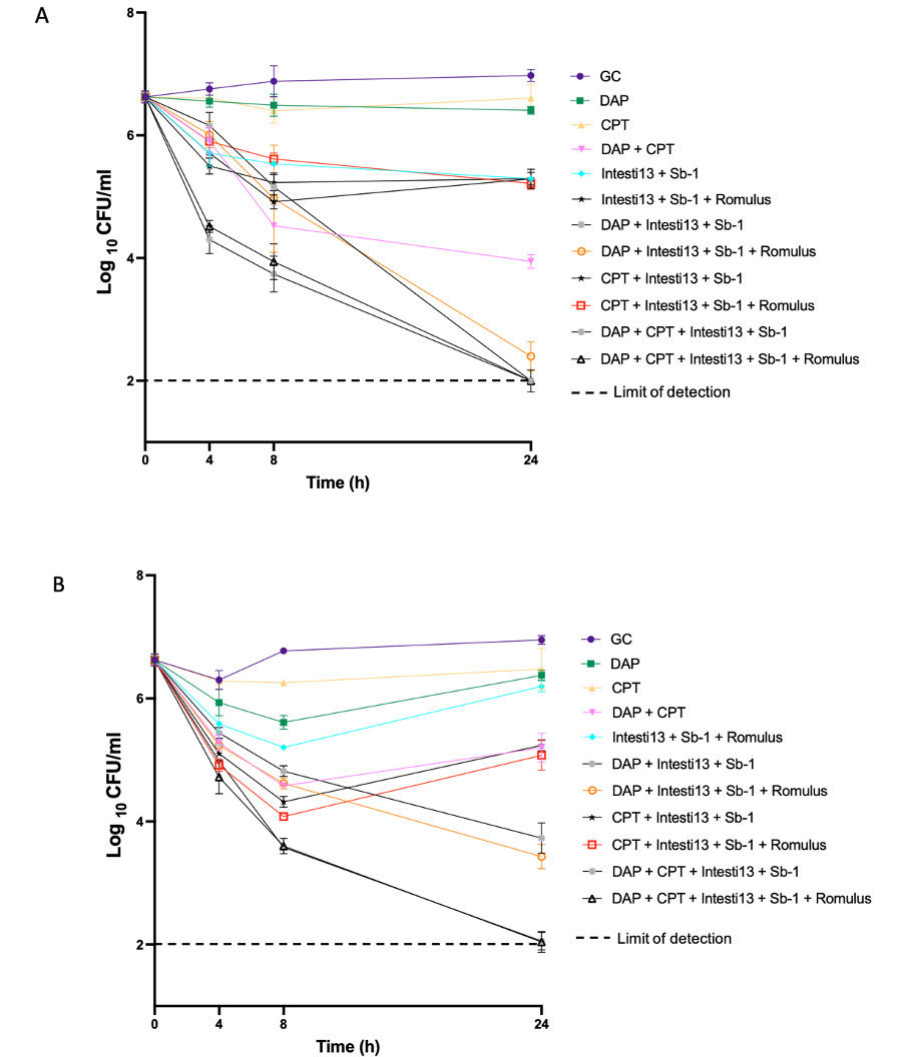
Bacterial quantification in 24-h biofilm time-kill experiments of daptomycin (0.5× MBIC), ceftaroline (free *C*_max_, 17), or daptomycin plus ceftaroline combined with phages Intesti13, Sb-1, and Romulus (each at a theoretical multiplicity of infection of 0.1) against DNS VISA and MRSA isolates (**A**) D712 and (**B**) 8014. The error bars indicate standard deviation of two replicate experiments. *P* values were determined using one-way analysis of variance and Tukey’s *post hoc* test.

To select the treatment regimens for PK/PD biofilm models, we performed 24-h biofilm time-kill experiments with D712 and 8014 to assess the potential for bactericidal killing over time with these agent combinations. Based on the CB MBIC data, we selected the following concentrations: DAP (0.5× MBIC), CPT (free *C*_max_ = 17 μg/mL), and phages Intesti13, Sb-1, and Romulus [each at a tMOI of 0.1 (2 × 10^8^ PFU/mL) based on modified CB MBIC results]. Bactericidal activity was defined as a ≥3log_10_ CFU/mL reduction from baseline at 24 h. Against both D712 and 8014, PAC containing DAP + CPT with 2-phage and 3-phage cocktails (Intesti13 + Sb-1 ± Romulus) demonstrated bactericidal activity with killing to detection limits (−Δ4.6log_10_ CFU/mL) (Fig. 2). Additionally, against D712, DAP with the 2-phage and 3-phage cocktails also demonstrated bactericidal activity (−Δ4.6 and 4.2log_10_ CFU/mL, respectively) while DAP with the 3-phage cocktail was bactericidal against 8014 (−Δ3.2log_10_ CFU/mL).

When we isolated surviving bacteria from TKAs run with strain 8014 and re-challenged them with phages, we identified treatment-emergent phage resistance when the 2-phage cocktail was used with DAP ± CPT; however, phage sensitivity was maintained at 24 h with the use of the 3-phage cocktail. Phage sensitivity was maintained at 24 h for D712 with both 2-phage and 3-phage cocktails.

### *In vitro* biofilm PK/PD model results

Table 1 summarizes the observed PK parameters of simulated regimens. Notably, PAC regimens used in D712 biofilm models utilized the 2-phage cocktail (Intesti13 + Sb-1) while the 3-phage cocktail (Intesti13 + Sb-1 + Romulus) was used in 8014 biofilm models. Our previous studies have examined both of these phage cocktails (13). Here, our 24-h TKA results revealed strain-specific differences in terms of which cocktail was associated with preventing the development of phage-resistant bacteria. To explore that observation further, we continued to use a different cocktail for each strain. Synergistic activity and bactericidal activity were defined as a ≥2log_10_ CFU/mL kill compared to the most effective antibiotic-only regimen and a ≥3log_10_ CFU/mL reduction from baseline at 168 h.

**TABLE 1:** Pharmacokinetic parameters for antibiotics used in *in vitro* biofilm models

Antibiotic	Target		Achieved		
	*ƒC*_max_ (μg/mL)	*T*_1/2_ (h)	*ƒC*_max_ (μg/mL)	*T*_1/2_ (h)	ƒAUC^*a*^_0-24_ (μg*h/mL)
DAP 10 mg/kg	11.3	8	12.1 +/-0.3	7.8 +/-0.0	138.8 +/- 2.3
DAP 8 mg/kg	9.8		10.9 +/- 0.1	8.1 +/- 0.0	128.9 +/-1.9
DAP 6 mg/kg	7.8		7.7 +/-0.1	8.1 +/- 0.0	92.0 +/- 1.9
CPT 600 mg	17	2.5	17.4 ± 0.3	2.6 ± 0.1	59.2 ± 0.4

^*a*^ AUC, area under the curve.

**Fig 3:**
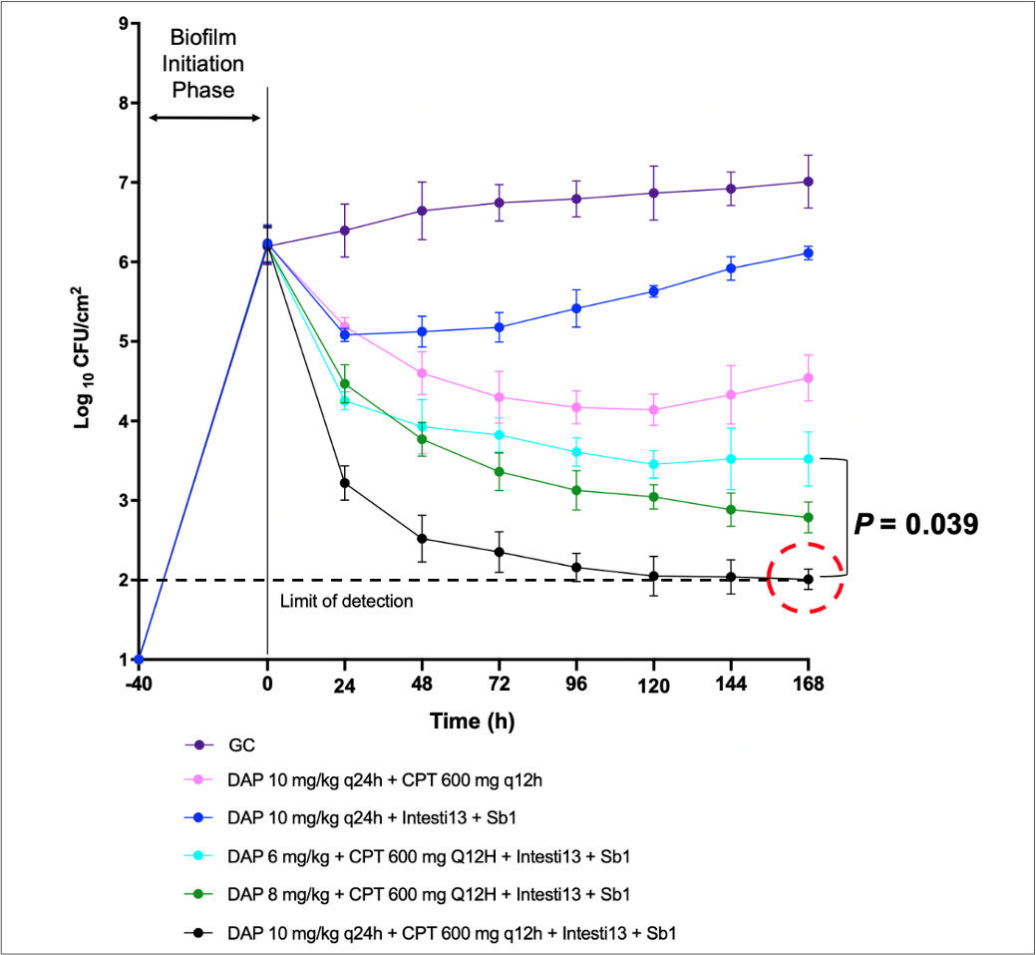
Efficacy of phage-antibiotic combinations in an *in vitro* PK/PD biofilm model against DNS MRSA strain D712.

**Fig 4:**
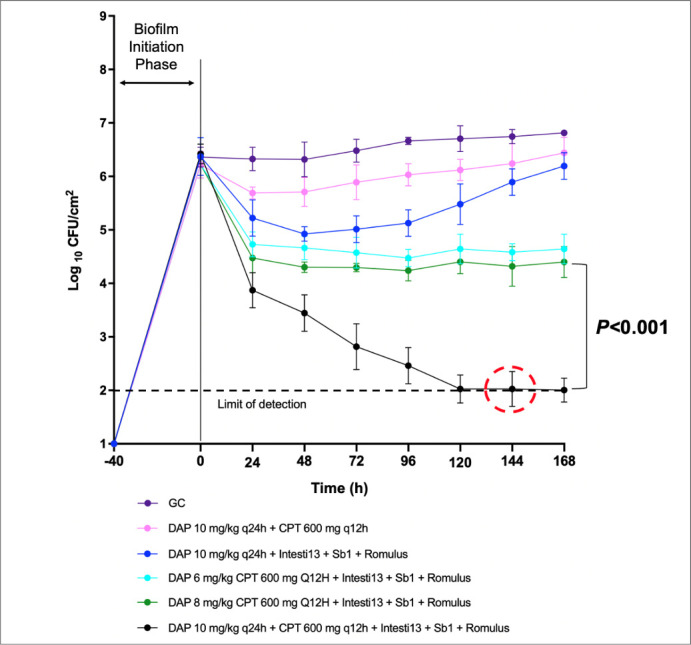
Efficacy of phage-antibiotic combinations in an *in vitro* PK/PD biofilm model against DNS MRSA strain 8014.

In 168-h biofilm models, DAP 10 + CPT + 2-phage cocktail (Intesti13 + Sb-1) had bactericidal activity against D712, causing a significant reduction in viability down to the detection limit of 2log_10_ CFU/cm^2^ (−Δ4.23log_10_ CFU/cm^2^; *P* < 0.001) (Fig. 3). The combination was also significantly more effective at bacterial killing compared to the dose de-escalation regimen of DAP 6 + CPT + 2-phage cocktail (*P* = 0.039). In 168-h biofilm models, bactericidal activity was identified with DAP + CPT + 3-phage cocktail (Intesti13 + Sb-1 + Romulus) against 8014, demonstrating a significant reduction in viability down to 2log_10_ CFU/cm^2^ (−Δ4.42log_10_ CFU/cm^2^; *P* < 0.001) (Fig. 4). The combination also demonstrated synergistic activity compared to the two-dose de-escalation regimens that included DAP 6 and 8 mg/kg (*P* < 0.001). Post-model antibiotic MBIC evaluations exhibited stabilization of DAP and CPT MBICs at 168 h (Table 2).

**TABLE 2:** Baseline and post-model minimum inhibitory concentration (MIC) values in planktonic and biofilm state

Strain	D712		8014	
Antibiotic	MIC (mg/L)	MBIC (mg/L)	MIC (mg/L)	MBIC (mg/L)
DAP	4	8	0.5	8
CPT	0.5	4	1	1

**Fig 5:**
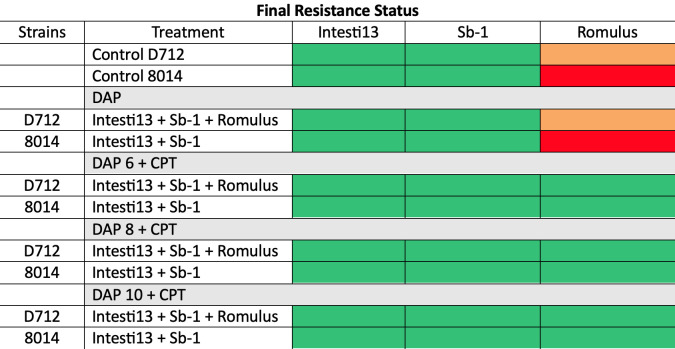
Phage sensitivity in control (baseline) and 168-h biofilm samples of DNS MRSA strains 8014 and D712 using the double drop method. Green, clear spot in double-drop method; orange, partial clearing within the spot; and red, resistant (phage activity not observed within the bacterial spot).

To evaluate whether there was a change in phage sensitivity in 168-h samples compared to baseline, the frequency of apparent resistance (FoR) (Fig. 5), followed by confirmatory testing of single FoR colonies in triplicate, was evaluated, as previously described, for phages used in the biofilm models (Intesti13, Sb-1, and Romulus) (16). Post-model evaluations of the three phages identified treatment-emergent phage sensitization for Romulus in biofilm models treated with PAC containing DAP + CPT dual therapy. No treatment-emergent phage resistance was identified in any of the models while models treated with DAP + phage demonstrated stabilization of phage activity across the board.

### Scanning electron microscopy results

**Fig 6:**
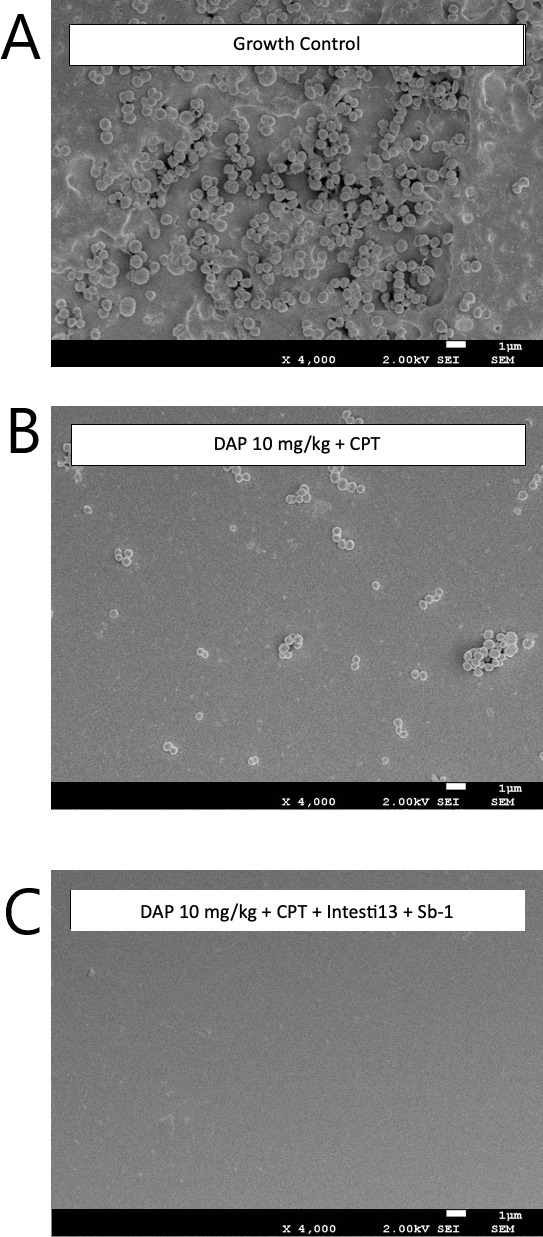
Scanning electron micrographs at ×4,000 magnification of polyurethane coupons with biofilm-embedded DNS VISA strain D712 after 168-h exposure to (**A**) neither antibiotics nor phage, (**B**) daptomycin plus ceftaroline, and (**C**) daptomycin 10 mg/kg plus ceftaroline with 2-phage cocktail Intesti13 plus Sb-1.

**Fig 7:**
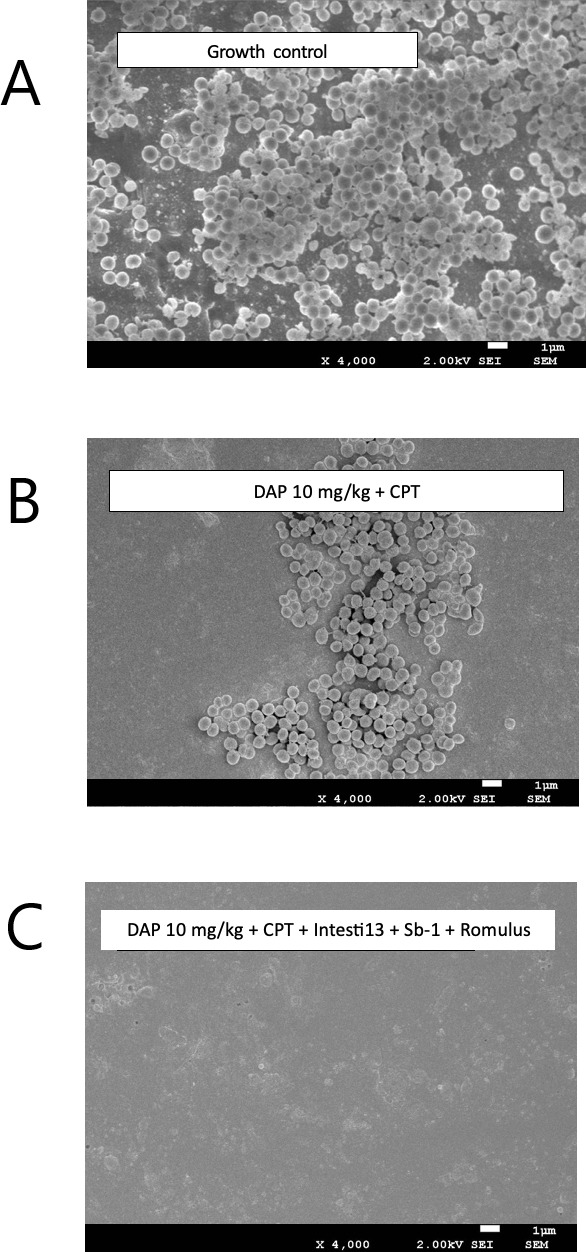
Scanning electron micrographs at ×4,000 magnification of polyurethane coupons with biofilm-embedded MRSA strain 8014 after 168-h exposure to (**A**) neither antibiotics nor phage, (**B**) daptomycin 10 mg/kg plus ceftaroline, and (**C**) daptomycin 10 mg/kg plus ceftaroline with 3-phage cocktail Intesti13 plus Sb-1.

The impact of antibiotic and phage exposure on established biofilm matrixes in the model was visualized by scanning electron microscopy (SEM) imaging of the polyurethane biofilm coupons. Biofilm development and the presence of embedded bacteria on the coupons prior to antibiotic exposure are shown in Fig. 6 and 7. Against D712, the antibiotic combination of DAP + CPT demonstrated some activity against MRSA-embedded biofilm, while the combination of DAP (at 6, 8, and 10 mg/kg) + CPT + the 2-phage cocktail (Intesti13 + Sb-1) was more effective at 168 h (all data not shown). Against 8014, some activity was identified with DAP 6 and 8 mg/kg + CPT + the 3-phage cocktail (Intesti13 + Sb-1 + Romulus); however, DAP 10 mg/kg + CPT + the 3-phage cocktails demonstrated substantially more activity.

## DISCUSSION

The emergence of biofilm-mediated medical device infections caused by MRSA has become a major clinical concern (1, 3, 4). Traditional antibiotics often fail to effectively eradicate biofilms due to unique properties of biofilm bacteria (17, 22). Phage-antibiotic combinations have shown promise in overcoming these challenges by exhibiting antibiofilm activity (13, 21). The use of PACs has been reported in single clinical cases (23–31) and discussed for use in clinical trials; however, systematic data about optimizing PACs are sparse. Furthermore, early stage-controlled trials listed on clinicaltrials.gov (phase 1-2) do not focus on DNS VISA or MRSA strains, leaving a critical knowledge gap in PACs against MRSA, an organism that often fails SOC antibiotic therapy.

In the current study, we evaluated the killing activity of standard of care antibiotics DAP and CPT, each at humanized concentrations, combined with phage cocktails containing phages Intesti13, Sb-1, and Romulus against two clinical biofilm-forming MRSA isolates, D712, a DNS VISA strain, and 8014, in 168-h biofilm PK/PD models. Our results demonstrate that the combination of DAP and CPT with 2-phage and 3-phage cocktails exhibited bactericidal activity against the biofilms. Specifically, DAP 10 mg/kg + CPT combined with 2-phage cocktail (Intesti13 + Sb-1) effectively reduced the bacterial viability of D712 biofilms to the detection limit. Similarly, the combination of DAP 10 mg/kg + CPT with a 3-phage cocktail (Intesti13 + Sb-1 + Romulus) showed significant reductions in bacterial viability in 8014 biofilms. These findings highlight the potential of PAC as a therapeutic strategy for biofilm-mediated MRSA infections.

One of the considerations in developing PAC therapies is the potential for antibiotic and/or phage resistance development. Our study assessed changes in antibiotic MBIC and phage sensitivity in post-model samples. Importantly, we found no changes in antibiotic MBIC compared to baseline, indicating that tested PAC did not induce antibiotic resistance. Moreover, the bacteria retained their sensitivity to phages even after 168 h in biofilm models, suggesting the stability of phage activity. These results provide valuable insights into the potential long-term effectiveness and sustainability of PAC therapy against MRSA biofilms and suggest that PACs have the potential to remain effective over extended treatment durations, which is expected to be important for combating chronic biofilm infections associated with indwelling medical devices.

While our study demonstrated early bactericidal effects up to 24 h in DAP 6 and DAP 8 conditions, a static effect was observed after 24 h, likely indicating the presence of persisters or bacteria tolerant to lower DAP concentrations. The concentration-dependent impact of DAP at 10 mg/kg/day appeared to overcome these persistence conditions. It is important to acknowledge that antibiotic and phage concentrations may vary at the infection site, such as in prosthetic joint infections. *In vivo* experiments will be essential to verify these preliminary findings and to address the question of metabolic stage and persistence, ensuring a comprehensive understanding of PAC efficacy in more clinically relevant settings.

We previously highlighted the importance of employing multiple phages, in the form of a phage cocktail, in combination with standard of care antibiotics to overcome phage resistance and enhance the overall *in vitro* efficacy of phage therapy against MRSA biofilms (13). The presence and genetic diversity in MRSA strains can result in the emergence of phage-resistant variants over time (32). By using a cocktail of phages, the likelihood of encountering phage-resistant strains is reduced, ensuring a broader spectrum of activity, and improving treatment outcomes (33). In our study, a different combination of phages was needed to prevent resistance in each of the two *S*. *aureus* strains. In future, we hope to explore what types of phage combinations could consistently reduce resistance development.

One hypothesis for how antibiotics can complement the action of phages is that they disrupt bacterial metabolism and weaken the biofilm structure, making the bacteria more accessible and thus, more susceptible to phage infection (34–36). Additionally, some phages degrade biofilm making biofilm-embedded organisms more accessible to antibiotics (37). The combination approach of antibiotics with phage cocktails can thus offer a synergistic effect, maximizing the changes of successful biofilm eradication and overcoming the challenges posed by MRSA biofilm infections. This aligns with our rationale for utilizing the 2-phage and 3-phage cocktails with standard of care anti-MRSA agents in the current study, aiming to maximize the changes of successful biofilm eradication and prevent the development of resistance.

In the current study, we also evaluated DAP dose de-escalation in PAC. Notably, although DAP is commonly used to treat serious MRSA infections, the optimal dosage for biofilm-associated infections is not well-established (38–40). Biofilms create a unique microenvironment that can impact antibiotic penetration and efficacy (6). Higher DAP doses may be required to effectively eradicate bacteria within the biofilm structure; however, reducing DAP doses may have advantages such as minimizing potential toxicity and optimizing drug utilization (41). Thus, we aimed to evaluate whether lower DAP doses (6 and 8 mg/kg) in PAC negatively impact bactericidal activity, antibiotic MBIC, and phage sensitivity against MRSA biofilms, compared to standard of care high DAP doses (10 mg/kg) in PAC. In the 168-h biofilm models, we identified that against DNS VISA strain D712, there was no significant difference in bactericidal activity with DAP 10 mg/kg + CPT with the 2-phage cocktail compared to DAP 8 mg/kg. However, DAP 6 mg/kg in PAC did exhibit significantly less bacterial killing compared to DAP 8 and 10 mg/kg. This contrasts with MRSA strain 8014 biofilm models, wherein PAC with DAP 10 mg/kg demonstrated significantly greater bacterial killing compared to PAC with DAP 6 or 8 mg/kg.

Future studies are warranted to further evaluate DAP dose-de-escalation schemes in PAC, including whether lower DAP doses in PAC allow phages to play a more prominent role in eradicating biofilm-associated MRSA. Phages have shown promise in targeting and disrupting biofilms; however, it remains to be determined whether lower antibiotic doses enhance the action of the phages and potentially reduce the selective pressure that can lead to the emergence of antibiotic-resistant strains. This combination approach may provide a more sustainable and effective treatment strategy against MRSA biofilm infections that also has the potential to minimize the risk of DAP dose-related toxicity (e.g., myopathy, eosinophilic pneumonia), improving patient safety and tolerability (42, 43).

Our study is not without limitations. While the 168-h *in vitro* models offer insight into PAC effectiveness against biofilm-embedded MRSA strains using humanized antibiotic doses, it is essential to acknowledge the inherent limitations of the *in vitro* studies herein, including sparse clinical data to help guide phage titer selection. For instance, available case reports of systemic phage used in the treatment of human infections lack evaluations of phage titer at the site of infection. This paucity of information leads to uncertainty about the optimal phage titer required to effectively target and combat infections at the site of interest. To address this challenge, we utilized *in vitro* modified CB MBIC and biofilm TKA as a proxy for combinatorial efficacy to screen various phage titer and antibiotic dilutions simultaneously to facilitate phage tMOI selection for use in 168-h biofilm models. Nevertheless, it is important to recognize that humanized models may not fully replicate the complexities of human physiology, including that *in vivo* phage clearance by the immune system could necessitate potentially higher phage titers in humans. Understanding the dynamics of phage clearance by immune cells could be instrumental in optimizing phage therapy for human use and mitigating any potential obstacles to treatment success. Therefore, investigations into the immunological aspects of phage therapy in human subjects will help inform *in vitro* studies as well as safe and effective treatment protocols moving forward. Another limitation of the *in vitro* studies herein is the abbreviated duration of therapy tested relative to extended durations of antibiotic therapy that is standard of care for biofilm-mediated medical device infections used clinically. Additionally, we utilized polyurethane coupons in the biofilm reactor to simulate a medical device-associated infection; however, medical devices may also contain components made of other polymers or metals and ceramics.

In conclusion, our study provides novel data highlighting bactericidal activity and synergistic activity of DAP and CPT combined with phage cocktails including phages Intesti13, Sb-1, and Romulus against DNS VISA and MRSA clinical isolates D712 and 8014 in biofilm PK/PD models with subsequent stabilization of antibiotic MBIC and phage sensitivity. Future studies are warranted to evaluate the efficacy of these PACs for longer durations of therapy that align with the standard of care for biofilm-mediated, medical device-associated infections. In particular, *in vivo* studies and clinical investigations in real-world settings are necessary to validate the findings from our *in vitro* models. Moreover, investigations into the optimal dosing regimens and treatment durations are warranted to optimize the therapeutic outcomes. Understanding the PK/PD properties of the PAC may contribute to the development of evidence-based treatment protocols for MRSA biofilm infections.

## MATERIALS AND METHODS

### Bacterial strains

Two clinical patient *S. aureus* isolates belonging to USA100ST5, D712 and 8014, were studied in this work. D712 (bioproduct PRJNA548818) (44) is a DAP-NS descendent of D592 (bioproduct PRJNA627464) (45), a DAP-susceptible MRSA strain collected from a patient with prolonged MRSA bacteremia (46, 47). 8014 (bioproduct PRJNA978117) (48) is a DAP-susceptible MRSA parent strain. Bacterial strain selection for the current study was based on previous data demonstrating that, out of a panel of MRSA strain pairs, biofilms of D712 and 8014 were the most resistant to killing by selected single phages (13).

### Bacteriophages

The selection of *S. aureus* bacteriophages Intesti13, Sb-1, and Romulus for this study followed methods outlined in prior research (13). In summary, the choice of the 3-phage cocktail for PAC was based on the phages’ distinct host ranges, evidence that spontaneous resistance to one phage did not necessarily lead to cross-resistance with other phages in the cocktails, minimal genome similarity, especially between at least two of the three phages, and demonstrated bacterial growth inhibition in *in vitro* biofilm conditions, both with and without antibiotics present (13, 20). The 2-phage cocktail is a subset of the previously tested 3-phage cocktail.

Intesti13 and Sb-1 originated from bacteriophage solutions procured from the Georgia Eliava Institute (Tbilisi, Georgia) and are close relatives of phage K, belonging to the *Kayvirus* genus (13, 49). Romulus was isolated in Belgium (50) and belongs to the *Silviavirus* genus (taxonomic classifications are current as of ICTV Master Species List #38). Sb-1, Intesti13, and Romulus were cultivated on *S. aureus* D712, ATCC 19685, and PS47, respectively.

The definition of phage MOI used herein refers to a particular phage titer (PFU/mL) added to a known total bacterial inoculum (*x*) in CFU/mL at a specific time point (*y*), which equates to a certain MOI if all cells in the biofilm were equally accessible.

### Antimicrobials

Daptomycin was obtained commercially from Xellia Pharmaceuticals (Buffalo Grove, IL, USA), and CPT analytical powder was obtained from AbbVie Inc. (North Chicago, IL, USA). Mueller-Hinton broth (MHB) (Difco, Detroit, MI, USA) supplemented with calcium and magnesium (50 and 12.5 µg/mL, respectively) was used for modified CB MIC, 24 h TKA, and 7-day biofilm models. Colony counts were performed on tryptic soy agar (TSA) plates (Difco, Detroit, MI, USA).

### Antibiotic susceptibility testing

MIC and MBIC testing of study antimicrobials was conducted prior to and following the completion of biofilm models in duplicate using manual broth microdilution at an initial microbial density of approximately 10^6^ CFU/mL according to Clinical and Laboratory Standards Institute (51). This method provided a quantitative assessment of the susceptibility of the target *S. aureus* strains to DAP and CPT by serially diluting them in MHB and identifying the lowest concentration at which microbial growth was inhibited.

### Biofilm modified checkerboard assay

We employed a customized checkerboard method to evaluate interactions between phages and antibiotics against MRSA isolates D712 and 8014, following previously established protocols (21). Briefly, we introduced MRSA-inoculated Glucose supplemented trypic soy broth (GSTSB) into a 96-well microtiter plate, covered it with a 96-pin lid, and incubated the setup at 37°C with agitation for 24 h to facilitate biofilm formation on the pins. Subsequently, after the incubation period, the pin lid was relocated to a separate 96-well microtiter plate containing diluted antibiotics and phages at 2-fold and 10-fold dilutions, respectively. When employing a checkerboard with two antibiotics, a constant MOI of phage (determined from single PAS checkerboard outcomes) was added once DAP and CPT dilutions were completed. The median volume per well was 200 µL. The plate was then subjected to 24-h incubation with agitation at 37°C, followed by spectrophotometric measurement at OD_570_. In each checkerboard, columns 11 and 12 were assigned as growth control and media control, respectively. Calculation of the FIC was performed for each checkerboard, where FIC indices of ≤0.5, 1–4, and >4 indicated synergy, additivity, and antagonism, respectively.

### Time-kill analyses

To assess bacterial growth inhibition, we conducted biofilm TKA in microwell plates over 24 h, in line with established protocols (13, 21, 40). Initially, each well contained four sterile 3-mm polystyrene beads immersed in a mixture of *S. aureus* at 6log_10_ CFU/mL and 1% GSTSB (1:9 ratio). Plates were incubated with agitation for 24 h at 37°C to encourage biofilm growth on the beads. The broth was replaced with calcium-supplemented and magnesium-supplemented MHB (2 mL). DAP and CPT were introduced to their designated wells at 0.5× MBIC and the maximum free, unbound serum drug concentration (free *C*_max_ = 17 µg/mL), respectively. Immediately after antibiotic addition, phages were introduced to appropriate wells at an MOI of 0.1 based on CB assay results. The median volume per well was 2 mL. At 0, 4, 8, and 24 h, a single bead was ascetically removed from each well. Biofilm removal from the bead was carried out for 6 min using intervals of vortexing and sonication (20 Hz, Bransonic 12 Branson Ultrasonic Corporation). Subsequent steps involved eliminating antibiotic and phage carryover through two rounds of centrifugation, supernatant removal, and appropriate dilutions in 0.9% saline. Samples were then plated on TSA and incubated at 37°C for 24 h before bacterial colony counting (Scan 1200, Interscience for Microbiology, Saint Nom la Breteche, France; detection limit of 10^2^ CFU/mL). Synergy and bactericidal activity were defined as a ≥2log_10_ CFU/mL kill compared to the most effective antibiotic-only regimen and a ≥3log_10_ CFU/mL reduction from baseline at 24 h. SPSS version 29.0 (IBM Corp., Armonk, NY, USA) software was utilized for statistical analysis. Assessment of significant differences between phage-antibiotic regimens in terms of bacterial killing metrics (i.e., log_10_ CFU/mL reductions from time 0 to 24 h) was accomplished through analysis of variance with Tukey’s *post hoc* test (*P* < 0.05).

### *In vitro* biofilm PK/PD model

To determine the impact of humanized DAP and CPT exposures and phage cocktails on biofilm-embedded D712 and 8014, we conducted a series of experiments with top-performing regimens in TKA in 168-h biofilm PK/PD models. Regimen selection for testing in biofilm models was based on 24-h biofilm TKA results. CDC biofilm reactor models (Biosurface Technologies, Bozeman, MT, USA) were established with biofilms grown on polyurethane coupons, following established procedures (52). In brief, a 40-h conditioning phase was conducted, involving incubation of bacteria-inoculated GSTSB at 37°C for 24 h, followed by 16 h of continuous GSTSB flow (13.3 mL/min) via peristaltic pumps (Masterflex; Cole-Parmer Instrument Co., Chicago, IL, USA). Subsequently, after the 40-h conditioning periods, the in-flow broth was switched from GSTSB to MHB with the subsequent flow rate being adjusted according to the *C*_max_, protein binding, and targeted adult half-life of DAP and CPT. For DAP plus CPT combinations models, supplemental DAP was added at an appropriate rate to compensate for the high flow rate required to simulate CPT clearance. Phages and antibiotics were then introduced into the reactor at predetermined intervals.

Regimen selection for testing in biofilm models was based on 24-h biofilm TKA results. In total, 12 separate 168-h *in vitro* PK/PD biofilm models were completed in duplicate, six for each D712 and 8014. Humanized doses of DAP 10, 8, and 6 mg/kg q24h and CPT 600 mg q8h were administered alone and in various combinations with the 2-phage and 3-phage cocktails (Intesti13 + Sb-1 ± Romulus, respectively) with each phage administered at a tMOI of 1 (4 × 10^11^ PFU/mL) dosed q12h based on preliminary CB MBIC and 24-h TKA results in biofilm state.

### PD analysis

From each model, two coupons were aseptically extracted at predefined time points (0, 4, 8, 24, 32, 48, 72, 96, 112, 136, 144, and 168 h). The coupons were rinsed with 10 mL of 0.9% saline to remove planktonic cells and transferred to a sterile tube containing 10 mL of 0.9% saline. To recover biofilm-embedded cells, an alternating sequence of three 60-s cycles of vortexing and sonication at 20 Hz (Bransonic 12; Branson Ultrasonics Corporation) was employed. Antibiotic carryover was nullified by appropriate dilutions of 0.9% saline. Diluted samples were then plated on TSA (easySpiral, Interscience for Microbiology, Saint Nom la Breteche, France, detection limit of 10^2^ CFU/mL), and incubated at 37°C for 24 h, followed enumeration of bacterial colonies (Scan 1200, Interscience for Microbiology, Saint Nom la Breteche, France).

### PK analysis

Pharmacokinetic samples were obtained in duplicate through the injection port of each model at 0,4,8 and 24 h for verification of target antimicrobial concentrations. Samples were stored at -80°C until analysis. DAP concentrations were measured by a validated high-performance liquid chromatography (HPLC) assay and CPT concentrations were determined by bioassay as previously described (53, 54). The half-life, free area under the curve (ƒAUC) and free peak concentration (ƒCmax) were determined by the trapezoidal method using PKAnalyst software (Version 1.10; MicroMath Scientific Software, Salt Lake City, UT).

### Scanning electron microscopy

SEM was performed on polyurethane coupons retrieved from each 7-day PK/PD biofilm model, following procedures outline in previous studies (Fig. 6 and 7) (38, 41, 55). After being retrieved during model sampling, the polyurethane coupons underwent a gentle wash with phosphate-buffered saline to eliminate nonadherent cells. Subsequently, they were fixed using a solution containing 2.5% glutaraldehyde and 2% paraformaldehyde in 0.1 M sodium phosphate buffer (pH 7.4). The samples were then subjected to two 15-min rinses in 0.1 M Sorenson’s buffer, followed by fixation in 1% osmium tetroxide for 20 min. Dehydration was carried out using a graduated series of ethanol, followed by immersion in hexamethyldisilane (HMDS) for four 15-min intervals. The coupons were allowed to air dry overnight after the fourth interval, with fresh HMDS applied to the coupons’ surface, which evaporated overnight in a fume hood. After 24 h, the SEM stubs hosted the mounted coupons, using a mixture of colloidal graphite and duco cement, sputter coating with gold was performed via Polaron sputter coated (Ernest F. Fullam Incorporated, Latham, NY, USA). Utilizing a JEOL JSM-7600F field emission scanning electron microscope, the samples were analyzed, and digital images were captures at ×4000 magnification employing Xstream imaging software to evaluate biofilm growth characteristics in relation to the coupon surface.
